# Janus Kinase-2 Mutation Associated Portal Vein Thrombosis Complicating Liver Cirrhosis and Hepatocellular Carcinoma

**DOI:** 10.31557/APJCP.2021.22.1.267

**Published:** 2021-01

**Authors:** Hatem Rabie, Warda Othman, Dalia M Elsabaawy, Eman E. Elshemy, Neamat Abdelmageed, Fatma A Khalaf, Hanan M Bedair

**Affiliations:** 1 *Departments of Clinical Pathology, National Liver Institute, Menoufia University, Egypt. *; 2 *Hepatology, National Liver Institute-Menoufia University, Egypt. *; 3 *Lecturer of Clinical Pharmacy, National Liver Institute, Menoufia University, Egypt. *; 4 *Hepatogastroentrology and Infectious Diseases, Faculty of Medicine for girls, AL-Azhar University, Egypt. *; 5 *Department of Biochemistry , National Liver Institute, Menoufia University, Menoufia, Egypt. *

**Keywords:** JAK2 RS V617F mutation, portal vein thrombosis, liver cirrhosis, hepatocellular carcinoma

## Abstract

**Background:**

Portal vein thrombosis (PVT) might be a catastrophic event complicating liver cirrhosis and hepatocellular carcinoma (HCC).

**Aim:**

role of *JAK2 RS V617F* mutation as a risk factor for PVT development in liver cirrhosis and HCC.

**Methods:**

A case control study conducted on 100 PVT patients (76 HCC and 24 liver cirrhosis) additionally, 100 healthy individuals used as a control group. PVT was diagnosed incidentally by Doppler ultrasound during routine follow-up HCC screening. *Prothrombin* *G20210A* mutation, *MTHFR* mutation, *Factor V Leiden *mutation (VFL), antithrombin III (ATIII), protein C, S, and antiphospholipid antibodies, along with *JAK2 RS V617F* mutation by real-time polymerase chain reaction all were analyzed.

**Results:**

Patients with PVT were significantly older (p<0.001), thrombocytopenic (p<0.001), with high alkaline phosphatase (p<0.001). *JAK2 RS V617F* mutation was found in 28/100 (28%) in idiopathic PVT complicating liver cirrhosis and hepatocellular carcinoma. Cases with positive *JAK2 rs V617F* mutation were significantly accompanied by protein S deficiency (P 0.03), LA absence (p 0.06), and high frequency of ascites (P 0.03). While, the *MTHFR heterozygous* mutation (p0.001), ATIII (P 0.02), and VFL (P 0.01) were more frequent with negative *JAK2 rs V617F* mutation. The comparison between demographic data and thrombophilic parameters in PVT cases revealed that no significant differences were recorded except for male gender, Diabetes Mellitus, splenomegaly significantly increased among HCC cases (p<0.05).

**Conclusions:**

*JAK2 rs V617F *mutation must be considered in any case of PVT with liver cirrhosis and hepatocellular carcinoma without identified thrombophilic risk factors, with potential considerations of evolving myeloproliferative disorders. New diagnostic and therapeutic implications are still awaited.

## Introduction

Portal vein thrombosis (PVT) is defined as a partial or complete obstruction of the portal vein by a clot resulting in impeded flow (Margini and Berzigotti, 2016), its prevalence ranges between 0.6% and 26% in cirrhotic and in about 35% in the setting of cirrhosis with hepatocellular carcinoma (HCC) (Tsochatzis et al., 2010; Chawla and Bodh, 2015). PVT may present with abdominal pain, portal hypertension, ascites, gastrointestinal bleeding, or mesenteric ischemia. Liver cirrhosis is among the most common acquired risk factors for portal vein thrombosis (PVT) and it is responsible for approximately 20% of all cases. Although PVT is most commonly seen in the setting of cirrhosis, many patients develop PVT in the absence of cirrhosis due to a combination of other prothrombotic factors (Fimognari and Violi, 2008). Also, HCC, acute pancreatitis, and intra-abdominal infection, systemic factors, including hypercoagulable states and sepsis, also pose an increased risk. The myeloproliferative neoplasms (MPNs) are associated with systemic prothrombotic states and are less frequently identified as the cause of PVT (Rao and Grosel, 2018).

PVT and Budd-Chiari syndrome (BCS) are relatively rare disorders; however, can be fatal if the underlying etiological factors are not diagnosed and treated. They have similar symptoms (splenomegaly, ascites, pain, and melena, etc.) and etiological factors such as factor *V Leiden (VFL)* mutation, prothrombin gene (*G20210A* mutation), protein C and protein S deficiency, and MPNs (Karaköse et al., 2015).

*Janus kinase 2 (JAK2)* is a non-receptor tyrosine kinase, which, upon ligand binding to specific cytokine receptors, is phosphorylated and activated, leading to regulation of gene expression involved in cell proliferation and survival. The acquired* JAK2* gene mutation on chromosome 9 (*JAK2 V617F*) is a mutation in G to T somatic mutation at nucleotide 1849 in exon 14, resulting in the substitution of valine to phenylalanine at codon 617, which triggers constitutive activation of downstream signaling and uncontrolled cell growth (Palumbo et al., 2019).

The acquired *JAK2* gene mutation on chromosome 9 (*JAK2 V617F*) is associated with polycythemia vera (PV) and other related MPNs (Tefferi and Gilliland 2005). The reported prevalence of the *JAK2* mutation has ranged from 65%-97% in polycythemia vera (PV) patients from Europe and North America, 23%-57% in patients with essential thrombocythemia (ET), and 35%-57% in primary myelofibrosis (PMF) patients (Yonal et al., 2012). The* JAK2 rs V617F *mutation has been implicated as an independent risk factor for portal and mesenteric vein thrombosis (Colaizzo, et al., 2007; Pan and Callahan, 2016).

A meta-analysis by Dentali et al. assessed the frequency of the *JAK2 RS V617F* is not only splanchnic vein thrombosis but also other thromboembolism including deep vein thrombosis and cerebral vein thrombosis. The *JAK2 rs V617F *mutation was noted to be more strongly associated with splanchnic vein thrombosis and it has been hypothesized that the *JAK2* mutation may affect the blood flow through the splanchnic venous bed (Dentali, et al., 2009). 

The *JAK2 rs V617F* mutation is a noninvasive molecular marker for occult MPNs and can be used for the diagnosis of latent MPNs presenting with thrombotic events, and the patients with idiopathic PVT or BCS showed that 20% had latent MPNs. In addition to this *JAK2* mutation, prothrombotic events were observed in a significant number of patients with splanchnic vein thrombosis. JAK2 gene analysis should be included in the research panel for BCS and PVT patients without cirrhosis (Karaköse et al., 2015).

The patients with venous thrombosis at other sites, the *JAK2 rs V617F* mutation was no more common than in the general population, and the frequency of the* JAK2 rs V617F *mutation was more strongly associated with PVT even in absence of other risk factors. The mechanisms underlying these findings have not been fully elucidated, therefore, the current study was designed. 

*Aim of the study*

This study aimed to detect the role of *JAK2 rs V617F *mutation in patients with chronic liver disease (cirrhosis and hepatocellular carcinoma) and clarify its value as a new risk factor for the development of portal vein thrombosis compared to other conventional thrombophilic factors.

## Materials and Methods

The current study was carried out on 100 patients with chronic liver disease (76 of them were HCC and 24 were with liver cirrhosis), who were attending the Hepatology and gastroenterology department at the National Liver institute-Menoufia University during the period from September 2018 to October 2019. In cirrhotic and HCC patients, PVT often is diagnosed incidentally by ultrasound during routine follow-up screening for HCC. Additionally, 100 apparently healthy individuals were enrolled in the study as a control group

All patients were subjected to complete history taking stressing on transient risk factors for venous thromboembolism (trauma, malignancy, surgery, pregnancy, oral contraceptive use, hormonal replacement therapy. A thorough clinical examination was done along with color Doppler Ultrasound for the evaluation of hepatic, inferior vena cava, and portal veins.

The research was approved by Ethical committee of National liver institute Menoufia University. An informed written consent was a prerequisite before enrollment in this study.

The inclusion criteria were: Age 18 years or older, all patients had liver cirrhosis or HCC confirmed with PVT proved by clinical, laboratory investigations and color Doppler Ultrasound examination.

The exclusion criteria: Any cause of PVT due to causes other than liver cirrhosis or HCC (such as Familial thrombophilia, thrombosis after surgical interference, Budd Chiari syndrome, or infections)

Peripheral blood samples were collected from all patients and controls and divided into two ethylene diamine tetra-acetic acid dipotassium salt (K2-EDTA) vacutainer tubes for CBC and RT-PCR and one citrate vacutainer tube for coagulation profile.

The following routine laboratory Investigations were done include Complete blood picture (CBC) using Sysmex automatic cell counter (Japan). PT, INR, and APTT using a fibrin timer analyzer. Liver and kidney function tests (ALT, AST, Albumin, total bilirubin, GGT, and ALP) were done by using the Integra 800 Auto analyzer (Roche-Germany).

Thrombophilia screening tests: prothrombin *G20210A *mutation, *MTHFR *mutation, *Factor V Leiden* mutation, serum level of antithrombin III, protein C, protein S, and antiphospholipid antibodies (lupus anticoagulant and anticardiolipin), the screening tests results were obtained from patient files.

Detection of *JAK2 rs V617F* mutation was done by Rotor gene Q real-time polymerase chain reaction (QIAGEN, GmbH-Germany). DNA extraction: Genomic DNA isolation from PBMCs was done using Gene JET Whole Blood Genomic DNA Purification Mini Kit (Thermo Scientific) according to Manufacturers’ instructions. The extracted DNA were tested to DNA purity, and finally stored at -80°C until subjected for real time PCR (Qiagen, 2016).

Detection of *JAK2* mutation was done by JAK2 Muta Screen Kit (catalog no. 673022, ipsogen, Germany) for the detection of the *JAK2 rs V617F/G1849T* mutation in genomic DNA. Two Taq Man probes are used, one is a perfect match to allele 1 sequence (e.g., mutant allele), and the other is a perfect match to allele 2 sequence (e.g., the wild-type allele). Each probe at its 5’ end is labeled with a different fluorescent dye (FAM or VIC), the reporter, and at the 3’ end contains a non-fluorescent quencher. During the extension phase of PCR, the perfectly matched probe is cleaved by the 5’→3’ exonuclease activity of Taq DNA polymerase, separating the reporter dye from the quencher and thus releasing detectable fluorescence. The probe that not perfectly matched will be displaced and no reporter dye will be released. The fluorescence signal (VIC or FAM) generated is collected at the end of the PCR (end-point) and immediately indicates the presence of the targeted sequence(s) in the sample (wild-type allele, mutated allele or both). Generation of fluorescence signal only with FAM dye indicate homozygous mutation while generation of fluorescence signal from FAM and VIC indicate heterozygous mutation (Qiagen, 2016).

*Statistical Methods*

Results were statistically analyzed using the statistical package of social sciences (SPSS 22.0, IBM/SPSS Inc., and Chicago, IL). Normally distributed continuous variables were expressed as mean and standard deviation (mean ± SD) while not normally distributed expressed as the median and interquartile range (IQR). Categorical data were expressed as frequency and percentage. For comparing continuous variables, ANOVA test was used when normality and homogeneity assumptions were met, instead, their non-parametric equivalent Kruskal-Wallis or Mann-Whitney tests were applied. The Chi-square test was used to compare categorical variables or alternatively, Fisher’s exact test was used when Chi-square assumptions were violated. The statistical significance was set at P-value <0.05.

## Results

Patients with PVT were found to be significantly older (p<0.001), thrombocytopenic (p<0.001), with higher levels of liver enzymes especially alkaline phosphatase (p<0.001). Comparison between PVT cases and controls regards demographic and routine laboratory variables revealed a statistically significant increase in the WBCs count, liver enzymes, bilirubin, INR, creatinine, urea, and AFP levels in PVT patients compared to healthy controls (p<0.001). In contrast, the levels of HB, platelet count, serum albumin were significantly decreased in patients with PVT (p<0.001). No significant differences were reported regards age and *MTHFR* mutation (p<0.24) (Table 1).

The distribution of J*AK2* mutation among idiopathic PVT cases with either HCC or liver cirrhosis was about 28/100 (28%), while secondary causes (cases with other thrombophilic risk factors) were found to be 72/100 (72%) (Table 2). The comparison between demographic data and thrombophilic parameters in PVT cases revealed that no significant differences were recorded except for male gender, DM, splenomegaly which is increased significantly increased among HCC cases (p vaules=0.02,0.001,0.03 respectively) (Table 3). 

Comparison between age and routine laboratory parameters in PVT cases shows a significant decrease in the levels of HB, WBCs, and platelet (p<0.001). While AST and AFP serum levels were significantly decreased in HCC cases with PVT compared to cirrhotic cases with PVT (p<0.001). No significant differences were reported regarding the other parameters (P>0.05) (Table 4). 

The cases with positive* JAK2 rs V617F* mutations were found to be significantly accompanied by protein S deficiency (P 0.03), LA absence (p 0.06), and high frequency of ascites (P= 0.03). While, the *MTHFR heterozygous* mutation (P=0.001), ATIII (P= 0.02) and VFL (P= 0.01) were more frequent with negative* JAK2 rs V617F* mutation. No significant differences were reported regarding the other thrombophilic risk factors, demographic and clinical data with *JAK2* mutation in patients with portal vein thrombosis (P>0.05) (Table 5). 

Non-significant differences were found between the CBC parameters, liver and kidney function tests, or serum AFP levels on comparing negative and positive *JAK2 rs V617F* mutation cases (P>0.05) (Table 6).

## Discussion

Patients with advanced liver cirrhosis and HCC represent a poor sector of patients gathering a dismal group of ailments that might end by PVT. The risk of PVT was said to increase in patients with liver cirrhosis by more than 7-fold increase above the general population (Ogren et al., 2006). Reports also had mentioned that the occurrence of only 1% of PVT in compensated cirrhosis which rises to 8%-25% in candidates for liver transplantation and 40% in the setting of HCC (Ponziani et al., 2012; Intagliata et al., 2018). These facts were evident in this study concluded when comparing cases with PVT and healthy control cases with a highly significant difference regarding complete blood picture, liver and kidney function tests and AFP 

Dealing with liver cirrhosis and HCC as a direct initiative of PVT is a fact but still in need of explanation. Chawla and Bodh, (2015) and Basit et al., (2015) found that the incidence of PVT among cirrhotic was 11.2%-16.6%, 20%-44% respectively, and in about 35% in HCC combined with cirrhosis. However, inherited or acquired thrombophilia were also seen in up to 20% of individuals with PVT with cirrhosis and HCC. Accordingly, liver cirrhosis either with or without HCC is not per se a cause of PVT and there might be a role for hidden thrombophilic disorders to be unveiled. 

*JAK2 rs V617F* mutation as a potential risk factor of PVT was studied added to other thrombophilic disorders in a cohort with liver cirrhosis and HCC. In the current study, *JAK2* mutation was found to be prevalent in 28/100 (28%) of cases with idiopathic PVT and secondary causes (cases with another factor mutation or acquired risk factors for thrombophilia) were about 72/100 (72%) of while cases. A finding which might imply a significant role of* JAK2* mutation in the development of PVT in patients with chronic liver disease even without overt myeloproliferative neoplasms (MPNs). 

The association between MPNs with *JAK2 rs V617F *mutation had been suggested to be linked to splanchnic vein thrombosis (Dentali et al., 2009). Therefore, a case of latent MPNs might be present irrespective of the normal thrombophilic or coagulation risk factors in hepatic patients.

Likewise, it might be the scenario in cases with PVT complicating liver cirrhosis and HCC with latent MPNs veiled by the devastating symptoms and signs of advanced chronic liver diseases. Hence, liver cell failure mortality in many instances might be attributed to evolving MPNs to death. Suppositions possibly would rationalize a lot of unexplained deteriorated chronic liver disease cases.

In agreement with our finding Amitrano et al., (2004) detected that inherited thrombophilic disorders in more than two-thirds of cirrhotic patients with PVT and the prothrombin gene *20210A* mutation were found to be associated with a more than a five-fold increased risk of developing PVT. Additionally, Amitrano et al., (2000) found that factor *V Leiden* mutation and *MTHFR- C677T* mutation are more frequently detected in cirrhotic patients with PVT compared to those without PVT.

Sharma et al., (2016) reported that low levels of AT III and proteins C and S have been reported in cirrhotic; but, it is not certain if this is true inherited thrombophilia or acquired from liver disease in contrast to these result a meta-analysis by Qi et al., (2013) showed that ATIII and proteins C and S levels are not significantly related to the development of PVT in cirrhosis. All these findings suggest a new way of dealing with PVT cases complicating liver cirrhosis and HCC at both therapeutic and diagnostic levels.

In this study, cases with positive *JAK2 rs V617F *mutation were significantly accompanying cases with a lessened level of protein S, and a high frequency of ascites. These results are in accordance with literature as PVT was said to be associating the advanced liver disease with severe ascites and liver cirrhosis with the documented reduced levels of protein S. While, the *MTHFR heterozygous *mutation, ATIII deficiency, and VFL were more frequent with negative *JAK2 rs V617F* mutation and no significant differences were reported regarding the other thrombophilia risk factors, demographic and clinical data with *JAK2 *mutation in patients with PVT. Therefore, a case of latent MPNs might be present irrespective of the normal thrombophilic factors or coagulation risk factors in hepatic patients, these results agreed with Dentali et al., (2009) who found that MPNs, specifically a *JAK2 rs V617F* mutation, are strongly associated with splanchnic vein thrombosis.

To our knowledge this is one of the first -if not the first- studies who investigated the relationship between *JAK2* mutation and PVT complicating liver cirrhosis and or HCC. The great number of PVT cases included in this cross-sectional study is also considered one of the strengths as most related studies relied on a smaller number of cases. However, more similar large-scale studies are mandated with further follow-up for the possibility of detection of MPNs development.

Conclusively, the diagnostic and therapeutic policies of dealing with PVT cases complicating liver cirrhosis had to change with the presumed role of thrombophilic disorders. Also, *JAK2 rs V617F* mutation testing essentially should be added to the list of PVT thrombophilic risk factors and should be investigated in every single case of idiopathic portal vein thrombophilia.

**Table 1:** Comparison between PVT Cases and Controls Regards Routine Laboratory Variables

	Parameters	PVT Cases (N=100)	Healthy controls (N=100)	p-value
Age (years)	Mean± SD	58.3± 6.3	49.3± 3.6	
	Min-max	33-68	35-61	0.32
HB (g/dl)	Median (IQR)	11.0 (3.0)	13.0 (1.0)	<0.001
	Min-max	7-16	11-17	<0.001
WBCs (x10^3^)	Median (IQR)	9.0 (4)	7.0 (2)	<0.001
	Min-max	3-19	4-10	<0.001
Platelets (x10^3^)	Median (IQR)	169.0 (160)	276.5 (149)	<0.001
	Min-max	50-519	182-441	<0.001
ALT (IU/L)	Median (IQR)	33.0 (39)	18.5 (12)	<0.001
	Min-max	6-735	10-29	<0.001
AST (IU/L)	Median (IQR)	52.0 (79)	19.0 (8)	<0.001
	Min-max	8-2248	10-29	<0.001
GGT (IU/L)	Median (IQR)	66.0 (102)	19.0 (5)	<0.001
	Min-max	12-534	10-32	<0.001
ALK ph (IU/L)	Median (IQR)	132.0 (77)	59.0 (23)	<0.001
	Min-max	44-990	31-99	0.24*
Albumin (g/dl)	Median (IQR)	3.0 (1.6)	4.0 (0.3)	<0.001
	Min-max	1-5	4-4.9	
T. Bilirubin (mg/dl)	Median (IQR)	2.0 (7.0)	0.4 (0.2)	<0.001
	Min-max	0.3-25	0.2-1	
INR	Median (IQR)	1.4 (0.5)	1.0 (0.03)	<0.001
	Min-max	1-2.3	0.9-1.1	
Urea (mg/dl)	Median (IQR)	64.0 (112)	32.0 (6.0)	<0.001
	Min-max	20.0-214.0	23.0-42.0	
Creatinine (mg/dl)	Median (IQR)	1.1 (0.7)	0.7 (0.1)	<0.001
	Min-max	0.6-4.6	0.6-0.99	
AFP (ng/ml)	Median (IQR)	72.2 (6647.3)	2.35 (1)	<0.001
	Min-max	0.73 - 150218.0	1.5-6.2	
MTHFR	Wild	58 (58)	66 (66)	0.24*
	Heterozygous	42 (42)	34 (34)	

LA, lupus anticoagulant; ACL, anticardiolipin; DAAS, direct acting antiviral drugs, FVL, factor V Leiden; MTHFR, methyl-tetrahydrofolate reductase; P>0.05 is non-significant; p <0.05: is significant; *Chi square test (x2) is used.

**Table 2:** The Distribution of *JAK2 *Mutation among Other Risk Factors of PVT

Causes	Parameters	PVT patients (N=100) No (%)
I. Idiopathic:	Cases with JAK2 mutation only	28 (28)
II. Secondary causes	Cases with another factor mutation or risk factors for thrombophilia	72 (72)
Secondary causes	Protein C deficiency	28 (28)
	Protein S deficiency	10 (10)
	ATIII deficiency	52 (52)
	MTHFR mutation:	
	Wild	58 (58)
	Heterozygous	42 (42)
	FVL mutation:	
	Wild	88 (88)
	Mutant	12 (12)
	Prothrombin G20210A mutation	
	Wild	96 (96)
	Mutant	4 (4)
	Antiphospholipid syndrome	
	ACL (Ig M)	8 (8)
	LA	10 (10)
	Tumor number in HCC cases (N=76)	
	Single	32 (42)
	Multiple	44 (57.9)
	Liver cirrhosis	24 (24)
	Ascites	70 (70)
	History of DAADs	44 (44)
	Diabetes mellitus	46 (46)
	Smoking	17 (17)
	Male gender	84 (84)

**Figure 1:**
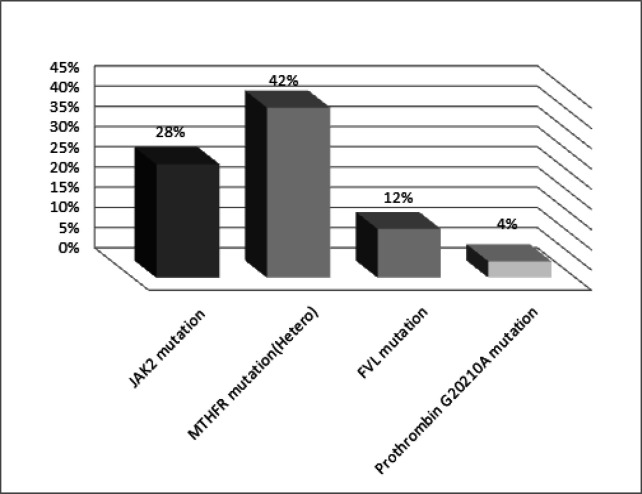
Genetic Mutation Frequencies of Thrombophilic Factors among PVT Patients

**Table 3:** Comparison between Demographic Data and Thrombophilic Parameters in PVT Cases

Parameters		HCC (N=76) N(%)	Cirrhosis (N=24) No(%)	P- Value
Age (years)	Median (IQR)	60.0 (8)	33.0 (13)	0.32
	Min-max	43-65	68-35	
Gender	Male	68 (89.5)	16 (66)	0.02*
	Female	8 (10.5)	8 (33.3)	
JAK2 RS V617F	Positive	22 (28.9)	6 (25)	0.7
Organomegaly	Hepatomegaly	30 (39.5)	8 (33.3)	0.58
	Splenomegaly	66 (86.8)	16 (66.7)	0.03*
Smoking	Positive	13 (17.1)	4 (16.7)	0.94*
Diabetes mellitus	Positive	28 (36.8)	18 (75)	0.001
Protein C	Deficiency	20 (26.3)	8 (33.3)	0.5
Protein S	Deficiency	8 (10.5)	2 (8.3)	1.00*
ATIII	Deficiency	40 (52.6)	12 (50)	0.82
FVL	Mutation	10 (13.2)	2 (8.3)	0.72*
G20210A	Mutation	4 (5.3)	0 (0)	0.57*
MTHFR	Heterozygous	36 (47.4)	6 (25)	0.05
LA	Positive	8 (10.5)	2 (8.3)	1.00*
ACL (IgM)	Positive	6 (7.9)	2 (8.3)	1.00*
Ascites	Absent	18 (23.7)	12 (50)	0.01
	Present	58 (76.3)	12 (50)	
History of DAAS	Receiving	42 (55.3)	10 (41.7)	0.27*
Child score	A	19 (26.3)	5 (33.3)	0.11
	B	22 (28.9)	9 (16.7)	
	C	35 (46.1)	10 (50)	

LA, lupus anticoagulant; ACL, anticardiolipin; DAAS, direct acting antiviral drugs; FVL, factor V Leiden; MTHFR,: methyl-tetrahydrofolate reductase; P>0.05 is non-significant; p <0.05: is significant; *Chi square test (x2) is used.

**Figure 2:**
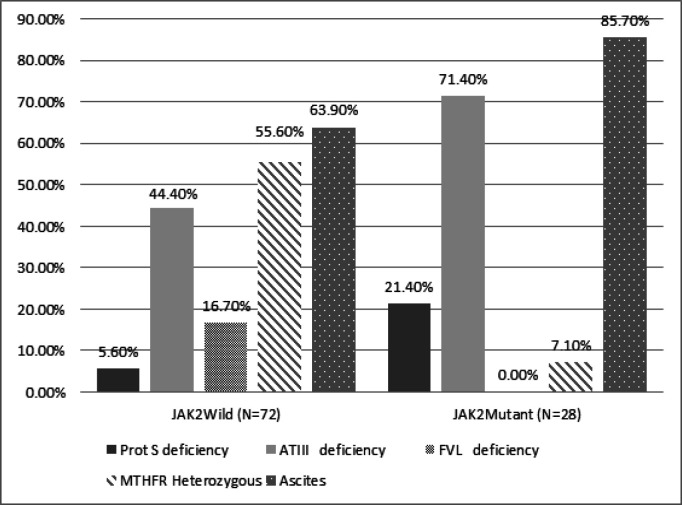
Significant Thrombophilic Factors Associated with *JAK2* Mutation

**Table 4:** Comparison between Age and Routine Laboratory Parameters in PVT Cases

Parameters		HCC (N=76)	Cirrhosis (N=24)	p-value
HB (g/dl)	Median (IQR)	11.0 (3)	9.5 (2)	0.02
	Min-max	7-16	8-14	
WBCs (x10^3^)	Median (IQR)	8.50 (4)	10.0 (3)	0.02
	Min-max	3-19	7-16	
Platelets (x10^3^)	Median (IQR)	149.0 (123)	265.0 (143)	0.002
	Min-max	57-519	50-354	
Neutrophils %	Median (IQR)	77.50 (19)	79.50 (13)	0.13
	Min-max	44-91	66-88	
lymphocytes%	Median (IQR)	14.00 (16)	11.50 (14)	0.18
	Min-max	4-38	4-28	
ALT (IU/L)	Median (IQR)	35.0 (42)	30.0 (32)	0.5
	Min-max	6-735	12-69	
AST (IU/L)	Median (IQR)	52.0 (99)	40.0 (48)	0.04
	Min-max	8-2248	11-126	
GGT (IU/dl)	Median (IQR)	62.50 (96)	61.50 (113)	0.75
	Min-max	15-534	12-456	
ALK ph (IU/dl)	Median (IQR)	129.0 (79)	132.0 (114)	0.96
	Min-max	44-990	61-537	
Albumin (g/dl)	Median (IQR)	2.60 (1.50)	2.70 (1.32)	0.72
	Min-max	1.00-4.90	1.20-3.60	
T. bilirubin (mg/dl)	Median (IQR)	2.0 (6.20)	2.19 (13.26)	0.58
	Min-max	0.40-23.80	0.30-25.20	
INR	Median (IQR)	1.46 (0.50)	1.51 (0.79)	0.47
	Min-max	1.00-2.33	1.06-2.23	
Urea (mg/dl)	Median (IQR)	50.5 (112.80)	76.50 (88.75)	0.93
	Min-max	22.0-214.0	20.0-151.0	
Creatinine (mg/dl)	Median (IQR)	1.10 (0.072)	1.135 (0.32)	0.82
	Min-max	0.60-4.60	0.60-3.19	
AFP (ng/dl)	Median (IQR)	339.5 (14543.4)	2.09 (21.84)	<0.001
	Min-max	1.10-150,218	0.73- 294.20	

P>0.05 is non-significant; p <0.05: is significant; Kruskal Wallis (U) test is used

**Table 5:** Comparison between Negative and Positive *JAK2 RS V617F* Mutation Cases as Regards Demographic and Clinical Data

Parameter		Wild JAK2 (No=72) No (%)	Mutant JAK2 (No=28) No (%)	P- Value
Age (years)	Median (IQR)	42.50 (19)	60.0 (4)	
	Min-max	33-68	50-65	0.64
Sex	Male	60 (83.3)	24 (85.75)	1
	Female	12 (16.7)	4 (14.3)	Fisher’s exact
Organomegaly	Hepatomegaly	30 (1.7)	8 (28.6)	0.22 (x^2^)
	Splenomegaly	58 (80.6)	24 (85.7)	0.54 (x^2^)
Smoking	Positive	9 (12.5)	8 (28.6)	0.13 (x^2^)
Hypertension	Present	21 (27.6)	7 (25)	0.27 (x^2^)
DM	Present	32 (44.4)	14 (50)	0.6 (x^2^)
Protein C	deficiency	18 (25.0)	10 (35.7)	0.28 (x^2^)
Protein S	deficiency	4 (5.6)	6 (21.4)	0.03* Fisher’s exact
ATIII	deficiency	32 (44.4)	20 (71.4)	0.02 (x^2^)
FVL	Mutation	12 (16.7)	0 (0)	0.01 (x^2^)
G20210A	Mutation	2 (2.8)	2 (7.1)	0.57 (x^2^)
MTHFR	Heterozygous	40 (55.6)	2 (7.1)	<0.001 (x^2^)
LA	Positive	10 (13.9)	0 (0)	0.06 * Fisher’s exact
ACL	Positive	6 (8.3)	2 (7.1)	1.00* Fisher’s exact
Ascites	Absent	26 (36.1)	4 (14.3)	
	Present	46 (63.9)	24 (85.7)	0.03 (x^2^)
History of DAAS	Positive	40 (55.6)	12 (42.9)	0.29 Fisher’s exact
Tumor number:	Single	24 (44.4)	8 (36.45)	0.51 (x^2^)
HCC cases (n=76)	Multiple	30 (55.6)	14 (63.6)	
Tumor size:	< 5 cm	30 (55.6)	16 (72.7)	0.16 (x^2^)
HCC cases (n=76)	=> 5 cm	24 (44.4)	6 (27.3)	
Child score:	A	17 (23.6)	7 (25)	
	B	23 (31.9)	8 (28.6)	0.12 (x^2^)
	C	32 (44.5)	13 (46.4)	

LA, lupus anticoagulant; ACL, anticardiolipin; DAAS, direct acting antiviral drugs; FVL, factor V Leiden; MTHFR, methyl-tetrahydrofolate reductase; P>0.05 is non-significant; p <0.05: is significant

**Table 6:** Comparison between Negative and Positive *JAK2 RS V617F* Mutation Cases as Regards Routine Laboratory Data

Parameter	Data	*JAK2 *Wild (N=72)	*JAK2*Mutant (N=28)	P-Value
HB (g/dl)	Median (IQR)	10.0 (3)	11.0 (2)	0.26(U)
	Min-max	7-16	7-14	
WBCs (x10^3^)	Median (IQR)	9.0 (5)	9.0 (4)	0.45(U)
	Min-max	3-19	3-14	
Platelets (x10^3^)	Median (IQR)	132.5 (174)	132.5 (117)	0.20(U)
	Min-max	50-519	57-511	
Neutrophils %:	Median (IQR)	75.5 (16)	81.5 (15)	0.06(U)
	Min-max	44-91	59-88	
Lymphocytes %	Median (IQR)	14.5 (14)	10.5 (9)	0.07(U)
	Min-max	4-38	4-30	
ALT (IU/L)	Median (IQR)	31.5 (42)	37.5 (29)	0.51 (U)
	Min-max	6-735	16-270	
AST (IU/L)	Median (IQR)	48.5 (82)	54.0 (54)	0.41(U)
	Min-max	8-2248	23-360	
GGT (IU/L)	Median (IQR)	67.0 (120)	53.5 (99)	0.81(U)
	Min-max	12-534	21-456	
ALK ph (IU/L)	Median (IQR)	123.5 (8)	140.5 (78)	0.66(U)
	Min-max	44-990	61-537	
Albumin (g/dl)	Median (IQR)	2.7 (1.5)	2.05 (1)	0.01(U)
	Min-max	1.00-4.9	1.6-3.8	
T.Bilirubin (mg/dl)	Median (IQR)	1.5 (6.4)	3.4 (7.2)	0.24
	Min-max	0.3-25.2	0.6 -24.6	
INR	Median (IQR)	1.4 (0.5)	1.6 (0.7)	0.04(U)
	Min-max	1.0-2.3	1.1-2.3	
Urea (mg/dl)	Median (IQR)	53.5 (108.8)	79.0 (112)	0.53(U)
	Min-max	20.0-214	24.0-180	
Creatinine (mg/dl)	Median (IQR)	1.1 (0.6)	1.1 (1.06)	0.90(U)
	Min-max	0.6-4.6	0.6-3.4	
AFP (ng/ml)	Median (IQR)	65.825 (8175.5)	152.6 (1380.9))	0.81(U)
	Min-max	73-100,000	1.2-150,218	

P>0.05 is non-significant; p <0.05: is significant; Kruskal Wallis (U) test is used
